# Examining the Role of Natural Mentors in the Relationship Between Childhood Abuse, Life Satisfaction, and Depression: Findings from Add Health

**DOI:** 10.1007/s40653-026-00873-8

**Published:** 2026-04-13

**Authors:** Savannah B. Simpson, Bailey R. Dow, Samuel D. McQuillin

**Affiliations:** Department of Psychology, University of South Carolina, 1512 Pendleton Street, Barnwell College, Columbia, SC 29208 USA

**Keywords:** Depression, Life Satisfaction, Childhood Abuse, Natural Mentors, Add Health

## Abstract

The current study investigated the impact of childhood abuse (i.e., physical abuse, sexual abuse, and neglect) on life satisfaction and depression in adulthood, while also examining the role of natural mentors. Natural mentors are nonparental adults from one’s social network (e.g., teachers, coaches, extended family, community members) who provide guidance and support. Data were drawn from the National Longitudinal Study of Adolescent Health (Add Health) across Waves I, III, and IV. The sample included individuals who indicated that they did (*n* = 11,434) and did not (*n* = 3,708) have a natural mentor at any time since the age of 14 years. Majority of participants identified as White (62.2%), followed by African American or Black (22.4%), Hispanic or Latinx (16.1%), Asian or Pacific Islander (7.9%), Other (8.9%), and American Indian or Native American (3.6%). Multigroup mediation analysis results indicated that all forms of childhood abuse negatively impacted life satisfaction, which in turn increased depressive symptoms in adulthood. The direct influence of childhood abuse on depression was also significant. Although life satisfaction significantly mediated the relationship between childhood abuse and depression, the presence of a natural mentor did not mitigate these effects. Thus, natural mentors did not buffer against the negative outcomes associated with exposure to childhood abuse. More research is needed to understand the interaction between natural mentoring relationships and childhood abuse.

## Introduction

Childhood abuse is a widespread public health problem that has enduring negative impacts on health and well-being (Brassard et al., 2020; Etter & Rickert, 2013). In fact, at least one in seven children within the United States (U.S.) have experienced abuse or neglect in the last year (Center for Disease Control and Prevention [CDC], (Center 2024); Finkelhor et al., 2015). When individuals experience abuse during the vulnerable developmental period of childhood, there are physical, psychological, and emotional ramifications that can continue into adulthood (Kendall-Tackett, 2002). Specifically, childhood abuse can negatively affect life satisfaction and contribute to depression in adulthood (Herrenkohl et al., 2012; Nurius et al., 2015). Emerging research on the protective impacts of positive childhood experiences suggests that safe, stable, and supportive relationships, such as those between young people and teachers, neighbors, and coaches (i.e., natural mentors), may mitigate the negative long-term impacts of abuse on health and well-being (Narayan et al., 2021). However, less is known about whether such natural mentoring relationships mitigate the long-term effects of childhood abuse on adult life satisfaction and depression. Therefore, using data from the National Longitudinal Study of Adolescent Health (Add Health), this study examined the relationship between childhood abuse and depression in adulthood through life satisfaction and whether that is contingent on having a natural mentor.

The current study explored child sexual abuse, physical abuse, and neglect, as these were the specific types of child abuse inquired about by Add Health and are three of the most common types of child abuse (CDC, 2024). Sexual abuse is defined as pressuring or forcing a child into sexual acts, such as fondling and penetration, while physical abuse involves physical force that may result in injury to the child, such as hitting, kicking, and burning (Child Welfare Information Gateway, 2022). Additionally, neglect is the failure to provide necessary care (e.g., food, clothing, medical care) that puts the child at risk of harm (Child Welfare Information Gateway, 2022). Children who are abused experience immediate negative effects on their well-being (e.g., interpersonal difficulties, depression, anxiety) that often endure throughout adulthood (Briere & Elliott, 1994; Norman et al., 2012). Some short-term consequences on well-being include physical injury (e.g., bruises, fractures, burns; DiScala et al., 2000; Leeb et al., 2011; Maguire, 2010) and mental health problems (e.g., attachment disorders, post-traumatic stress, mood disorders, externalizing behaviors; Leeb et al., 2011). In fact, around 90% of children who experience sexual abuse and 50% of children who experience physical abuse show symptoms of post-traumatic stress disorder (Leeb et al., 2011).

These mental health challenges tend to persist long after the incident(s) of abuse and into adulthood (De Bellis, 2001; Strathearn et al., 2020). Adolescents who experienced childhood abuse are more likely to exhibit externalizing, violent behaviors (e.g., physical fighting, weapon carrying, threatening, property offenses), deliberate self-harm (e.g., suicide attempts, eating disorders, non-suicidal self-injury), and depressive symptoms (Leeb et al., 2011; Strathearn et al., 2020). These externalizing and internalizing behaviors continue across development and can manifest into clinical disorders in adulthood. Furthermore, adults who experienced childhood abuse have a greater likelihood of being diagnosed with a personality disorder, mood disorder, or post-traumatic stress disorder than those who did not experience childhood abuse (Leeb et al., 2011; Strathearn et al., 2020).

The long-lasting impacts of childhood abuse also influence broader aspects of mental health and well-being, such as quality of life. Those who have experienced childhood abuse tend to report a lower quality of life (Corso et al., 2008). Life satisfaction is a personal cognitive appraisal of the quality of one’s life based on subjective well-being (Pavot & Diener, 2008; Proctor et al., 2009). Researchers have illustrated how childhood abuse can adversely affect life satisfaction (Herrenkohl et al., 2012; Nurius et al., 2015) and contribute to depression in adulthood (Negele et al., 2015). Individuals who experience childhood abuse report lower levels of current life satisfaction which tends to persist throughout adulthood (Herrenkohl et al., 2012; Park et al., 2023; Mosley-Johnson et al., 2019). Similar patterns have been found with depression. Researchers have shown that childhood abuse is positively correlated with depression (Berber Çelik & Odacı, 2020; Goldberg, 1994; Nelson et al., 2017). Those who report a history of childhood abuse tend to experience more severe depression symptoms compared to those with no abuse history (Cukor & McGinn, 2006). Together, life satisfaction and depression can be conceptualized through the dual-factor model of mental health (Suldo & Shaffer, 2008).

The dual-factor model of mental health suggests that mental health includes both positive (e.g., subjective well-being) and negative (e.g., pathological symptoms) psychological experiences (Kelly et al., 2012; Suldo & Shaffer, 2008). Child abuse negatively impacts both positive and negative psychological experiences. Individuals who experience child abuse often report lower levels of subjective well-being and life satisfaction in adulthood (Tanaka et al., 2015) and more anxiety and depressive symptoms over the life course (Lindert et al., 2014). However, this is not always the case, as there are more nuances to mental health. It is possible for individuals to feel happy and live meaningful lives while experiencing mental health symptoms. Additionally, an absence of mental health symptoms does not necessarily equate to feelings of happiness and life satisfaction (e.g., Keyes, 2007; Lecci et al., 1994). Indeed, individuals with greater social support are more likely to have average to high levels of subjective well-being regardless of their psychopathology symptoms (Kelly et al., 2012). In light of psychopathology, social support appears to be an important factor in maintaining mental health.

Resilience literature has posited the importance of social support as a protective factor in the face of adversity (Buchanan et al., 2023; Panagou & Macbeth, 2024). Emerging research on the protective impacts of positive childhood experiences indicates that safe, stable, nurturing relationships, such as those between young people and teachers, neighbors, and older peers, may mitigate the negative long-term impacts of abuse on health and well-being (Narayan et al., 2021; Park et al., 2023). Indeed, positive childhood experiences like this safe, stable social support have been shown to independently provide long-term protection against poor mental health outcomes even when adverse childhood experiences are co-occurring (Xu et al., 2022; Bethell et al., 2019; Narayan et al., 2018). Specifically, those who experience more positive childhood experiences are less likely to develop adult depression or other mental health challenges (Bethell et al., 2019). Safe, stable, and supportive relationships may come from organized, formal activities, like formal youth mentoring programs. For example, children who have been abused and placed in foster care report fewer mental health problems and higher life quality after participation in a formal mentoring program (Taussig & Culhane, 2010). Supportive relationships can also come from informal, daily interactions (e.g., extended family members, teachers, coaches) from one’s existing social network (i.e., natural mentors).

Natural mentoring relationships are typically defined as informal, supportive, and caring connections with nonparental adults that develop organically, independent of any pre-established matching or formal program structures (DuBois and Silverthorn 2005a). Such relationships often emerge in everyday contexts—including schools, workplaces, communities, and extended families—and may involve teachers, coaches, neighbors, or other adults who provide ongoing guidance, encouragement, and support (Deutsch et al., 2020). These naturally formed relationships have been proposed as important sources of resilience (Hurd & Zimmerman, 2010; Raposa & Hurd, 2021), potentially buffering the negative effects of early adversity on later well-being. One recent study found that those who reported caregiver physical abuse were more likely to identify a natural mentor than those without such abuse histories (Weber Ku et al., 2021). However, these mentoring relationships tended to be less close, shorter in duration, and characterized by less frequent contact compared to those reported by individuals without abuse histories. Taken together, these findings suggest that while survivors of physical abuse may be somewhat more likely to develop natural mentoring connections, the quality and stability of those relationships may be diminished.

The present study extends this line of work by examining whether the presence of a natural mentor is associated with adult well-being—specifically, life satisfaction and depression—among individuals who experienced childhood abuse. Understanding this relationship is critical, as individuals exposed to childhood trauma are at elevated risk for poor mental health and lower life satisfaction in adulthood (Tanaka et al., 2015; Lindert et al., 2014). Yet, supportive relationships, such as those with natural mentors, may foster resilience and adaptive coping, potentially mitigating these risks (Weber Ku et al., 2021). Recognizing the potential influence of natural mentoring on long-term outcomes is essential for informing prevention, early intervention, and treatment services. While resilience and social support frameworks (e.g., Cohen & Wills, 1985; Masten, 2018) are central to understanding adaptive functioning after adversity, they may not fully capture the contextual complexities of natural mentoring relationships. Indeed, critiques of resilience theory highlight that traditional models often overemphasize individual traits or coping skills while underestimating the social and structural conditions that shape opportunities for resilience (Van Breda, 2018). These contextual factors also influence youth’s access to supportive relationships. For example, individuals who face greater socioeconomic or systemic barriers may have fewer opportunities to form and sustain natural mentoring relationships despite their potential benefits, and even when these relationships are formed, they often differ in their function and scope (e.g., practical support rather than career advice; Raposa et al., 2018). More recent conceptualizations view resilience as a dynamic, relational process embedded within broader ecological contexts, which is particularly relevant for understanding natural mentoring relationships that are built outside formal systems of support. Building on this perspective, it is important to consider how naturally occurring mentoring relationships—often unstructured and shaped by contextual factors—may function as protective mechanisms that buffer the long-term effects of childhood abuse on well-being. Therefore, the current study examined the relationship between childhood abuse (i.e., sexual abuse, physical abuse, and neglect) and depression in adulthood through life satisfaction, and whether this relationship varies according to the presence of a natural mentor. We hypothesized that individuals with a natural mentor, compared to those without one, would report fewer negative effects of childhood abuse on life satisfaction and, in turn, lower depressive symptoms.

## Method

### Participants and Procedure

Data were drawn from Wave I, III, and IV of the National Longitudinal Study of Adolescent Health (Add Health). These specific waves were selected because they include all key constructs required for the present analyses: Wave I provides baseline demographic and contextual variables; Wave III includes retrospective reports of childhood abuse, neglect, and natural mentoring relationships, as well as participants’ self-reported life satisfaction; and Wave IV assesses adult depressive symptoms. Add Health is a nationally representative longitudinal study on adolescent health into adulthood, in which researchers measured social, economic, psychological, and physical well-being, and contextual factors (e.g., family, neighborhood, community, school, relationships) across multiple time points (Harris et al., 2019). Add Health data were collected across five waves from a nationally representative sample of U.S. adolescents in middle and high school, following participants into adulthood (last data collection in 2018; aged 33–43). In Wave I, data were collected from 1994 to 1995 and included 20,475 participants who were interviewed about topics on demographics, peer and familial relationships, education, and health status. Data collection in Wave III occurred from 2001 to 2002 and included 15,197 participants who completed the second follow-up interview. In Wave IV, 15,701 participants were re-interviewed from 2008 to 2009. Only participants who completed Wave I, III, and IV and indicated if they did or did not have a natural mentor were included in this study (*n* = 15,142). Table 1 contains descriptive statistics for demographics. Add Health participants provided written informed consent for participation in all aspects of Add Health in accordance with the University of North Carolina School of Public Health Institutional Review Board guidelines that are based on the Code of Federal Regulations on the Protection of Human Subjects 45CFR46: https://www.hhs.gov/ohrp/humansubjects/guidance/45cfr46.html.

**Table 1:** Participant Demographic Characteristics

Variable	No Natural Mentor (*n* = 3,708)	Natural Mentor (*n* = 11,434)
Biological Sex		
Female	1844 (49.7%)	6128 (53.6%)
Male	1857 (50.1%)	5286 (46.2%)
Race/Ethnicity		
African American or Black	892 (24.1%)	2507 (21.9%)
White	2092 (56.4%)	7324 (64.1%)
Hispanic or Latinx	799 (21.5%)	1640 (14.3%)
Asian or Pacific Islander	304 (8.2%)	888 (7.8%)
American Indian or Native American	130 (3.5%)	412 (3.6%)
Other	454 (12.2%)	900 (7.87%)
	*M(SD)*	*M(SD)*
Family Income in 1994	41,400 (43,300)	47,500 (46,900)

## Measures

### **Demographic Characteristics**

At Wave I, participants reported their race, ethnicity, and biological sex, while participants’ primary caregivers reported their household income before taxes in 1994.

### **Natural Mentor**

Participants were asked in Wave III, “Other than your parents or step-parents, has an adult made an important positive difference in your life at any time since you were 14 years old?” This question was used to indicate if participants did or did not have a natural mentor—responses were dichotomized (1 = natural mentor; 0 = no natural mentor).

### Childhood Abuse

In Wave III, childhood abuse was examined by asking three questions about (1) neglect (“How often had your parents or other adult care-givers not taken care of your basic needs, such as keeping you clean or providing food or clothing?”); (2) physical abuse (“How often had your parents or other adult care-givers slapped, hit, or kicked you?”); (3) sexual abuse (“How often had one of your parents or other adult care-givers touched you in a sexual way, forced you to touch him or her in a sexual way, or forced you to have sexual relations?”). Responses were coded on a 6-point scale ranging from 0 = “Never” to 5 = “More than ten times.”

### **Life Satisfaction**

 Participants reported on their life satisfaction in Wave III (“How satisfied are you with your life as a whole?”) using a 5-point Likert scale ranging from 1 = “Very dissatisfied” to 5 = “Very satisfied.”

### **Depression**

At Wave IV, the 10-item Center for Epidemiologic Studies Depression Scale (CES-D; Andresen et al., 1994) was used to assess for depressive symptoms in the past week. Participants responded on a 4-point scale ranging from 0 = “Never or rarely” to 3 = “Most of the time or all of the time.” Higher scores suggest greater severity of depressive symptoms (𝛼 = 0.84).

### Data Analysis

**Fig. 1:**
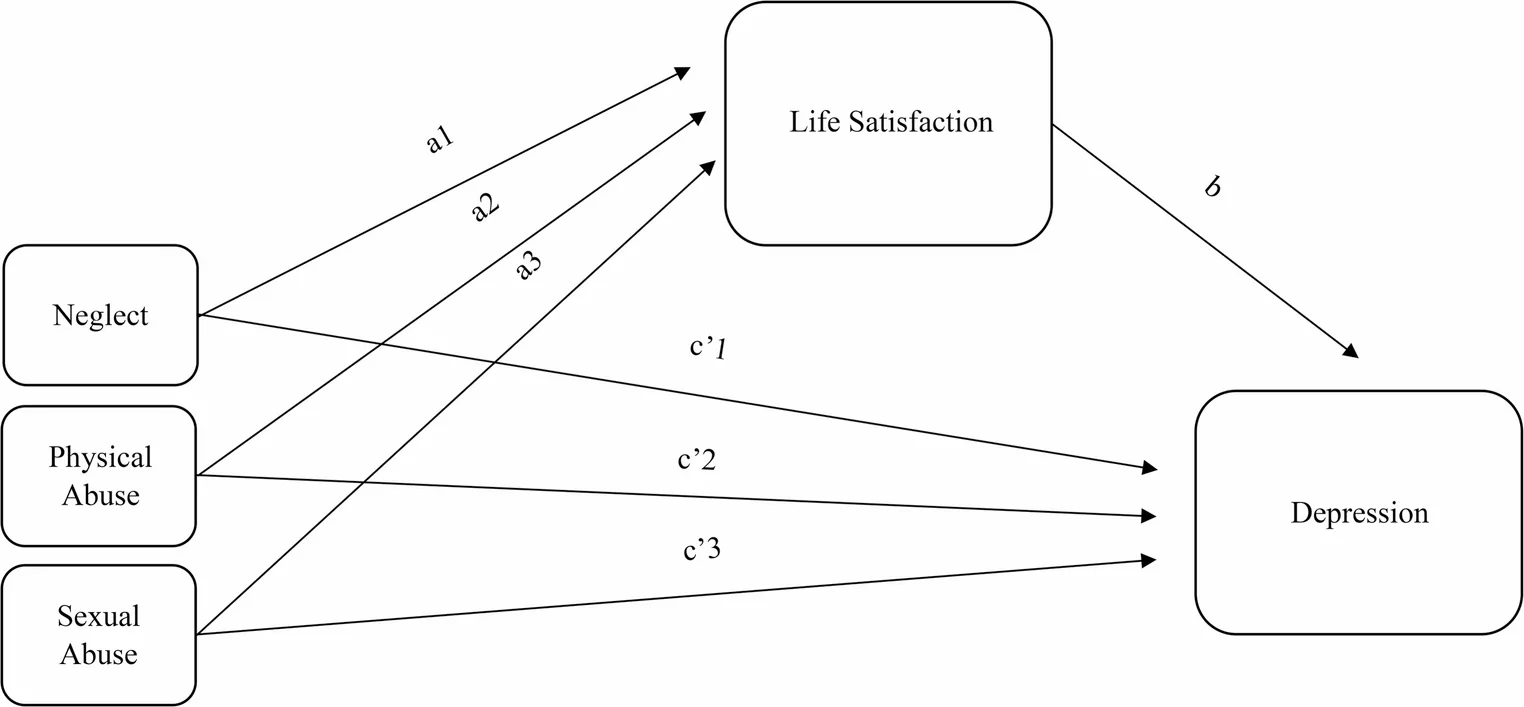
Life Satisfaction as a Mediator of the Relationship Between Childhood Abuse and Depression for those with and without a Natural Mentor

Multigroup mediation analysis was used to test if children with a natural mentor, compared to children without a natural mentor, report less negative effects of childhood abuse on life satisfaction and subsequently less depressive symptoms. In this model, we are interested in the *a* path (i.e., the influence of childhood abuse on life satisfaction), the *b* path (i.e., the influence of life satisfaction on depression controlling for childhood abuse), and the a*b path (i.e., the indirect effect of childhood abuse on depression through life satisfaction) for those with and without a natural mentor, as depicted in Fig. 1. Analyses were conducted using Mplus version 8.2 (Muthén & Muthén, 1998–2017). Missing data were handled using full information maximum likelihood (FIML) reducing sample size from 15,142 to 15,139 for analysis. Model fit was examined using the 𝜒^2^ test, the root mean squared error of approximation (RMSEA), the comparative fit index (CFI), and the standardized root-mean-square residual (SRMR). Good model fit was indicated by RMSEA < 0.05, CFI ≥ 0.90, and SRMR < 0.08 (Hu & Bentler, 1999), although the 𝜒^2^ value was significant. As mentioned before, we tested statistical mediation using the product of coefficients (i.e., indirect effects in Mplus) and tested group differences (i.e., natural mentor versus no natural mentor) using multigroup mediation, wherein differences between the groups’ mediated path was tested using the model constraints function in Mplus by creating a parameter representing the differences between the product of coefficients (i.e., a*b for those who were mentored subtracted from a*b for those who were not). This analysis tests whether mediated paths differ on the basis of group. 

## Results

All forms of childhood abuse influenced life satisfaction (paths *a1*, *a2*, and *a3*), life satisfaction influenced depressive symptoms (i.e., the *b* path), and childhood abuse influenced depression (*c’1*, *c’2*, and *c’3*) later in life (see Tables 2 and 3 for results). Moreover, the mediated effect of life satisfaction for all forms of childhood abuse (a1*b, a2*b, and a3*b) was statistically significant. However, the presence or absence of a natural mentor did not significantly influence the effects of any form of childhood abuse on life satisfaction or the mediated effect of life satisfaction on depression. Thus, contrary to our hypothesis of the presence of a natural mentor serving as a protective factor against childhood abuse, children were similarly likely to have low life satisfaction and subsequent depressive symptoms regardless of having a natural mentor or not.

**Table 2:** Test of Difference Between Paths and Mediated Effects for those with and without a Natural Mentor

Table 2 Mediated Effects Between Groups						
	No Natural Mentor (*n* = 3,708)			Natural Mentor (*n* = 11,434)		
Pathways	*b* (*SE*)	*z*	*p*	*b* (*SE*)	*z*	*p*
Direct effect of child abuse on life satisfaction (*a*)						
Neglect (*a1*)	0.042 (0.014)	2.974	0.003	0.025 (0.008)	3.199	0.001
Physical Abuse (*a2*)	0.075 (0.011)	7.060	0.000	0.080 (0.005)	15.686	0.000
Sexual Abuse (*a3*)	0.072 (0.026)	2.819	0.005	0.028 (0.013)	2.086	0.037
Direct effect of life satisfaction on depression (*b*)	1.472 (0.101)	14.505	0.000	1.445 (0.056)	25.655	0.000
Direct effect of child abuse on depression, residualized for the mediated path (*c’*)						
Neglect (*c’1*)	0.333 (0.087)	3.816	0.000	0.177 (0.047)	3.740	0.000
Physical Abuse (*c’2*)	-0.089 (0.065)	-1.371	0.170	0.153 (0.031)	4.935	0.000
Sexual Abuse (*c’3*)	0.701 (0.153)	4.570	0.000	0.252 (0.079)	3.183	0.001
Indirect effect (mediated effect) of child abuse on depression through life satisfaction (a*b)						
Neglect (a1*b)	0.062 (0.021)	2.913	0.004	0.037 (0.012)	3.175	0.001
Physical Abuse (a2*b)	0.111 (0.017)	6.345	0.000	0.116 (0.009)	13.397	0.000
Sexual Abuse (a3*b)	0.106 (0.038)	2.768	0.006	0.040 (0.019)	2.078	0.038

**Table 3:** Test of Difference Between Paths and Mediated Effects for those with and without a Natural Mentor

*a* and *b* Path Differences	*b* (*SE*)	*z*	*p*
Neglect (*a1*)	− 0.017(0.016)	-1.03	0.301
Physical Abuse (*a2*)	0.005(0.012)	0.439	0.661
Sexual Abuse (*a3*)	− 0.044(0.029)	-1.541	0.123
Life Satisfaction (*b*)	− 0.026(0.116)	− 0.225	0.822
Mediated Effects Differences			
Neglect (a1*b)	-0.025 (0.024)	-1.049	0.294
Physical Abuse (a2*b)	0.006 (0.020)	0.284	0.776
Sexual Abuse (a3*b)	-0.066 (0.043)	-1.541	0.123

## Discussion

Our findings add to the existing literature and highlight the inconsistent effects of having a natural mentor as a viable prevention tool in children’s well-being. Prior research has indicated that supportive, mentoring relationships may buffer against negative outcomes for “at-risk” youth (Brown & Schillington, 2017; Greeson, 2013; Prowell & Williams, 2021; Southwick et al., 2007; Rhodes et al., 2006). For example, individuals who experienced childhood neglect were less likely to feel depressed in adolescence when they had a satisfying relationship with a mentor (Lee et al., 2021). Further, individuals who experienced childhood sexual abuse were more likely to have higher social and emotional well-being when they had a mentor (Prowell & Williams, 2021). As such, mentors have been identified as a source of resilience for youth. Community organizations have echoed this in their mentoring initiatives (e.g., Adverse Childhood Experiences [ACEs] mentoring) that focus on fostering mentoring relationships with “at-risk” youth to support their resilience across development (Parker & Larkin, 2022).

The extant literature guided us in postulating that natural mentoring relationships could protect against the negative outcomes associated with childhood abuse. However, our findings indicate that the presence of a natural mentor did not protect individuals against the negative outcomes associated with childhood abuse, particularly depression and lower life satisfaction. This provides contrary evidence to the idea that mentors can mitigate negative youth outcomes through caring and supportive relationships. While mentors provide youth with supportive, stable relationships, those relationships alone may not be enough to significantly mitigate the negative outcomes of childhood abuse. One possible explanation for this pattern is that the *function* and *meaning* of natural mentoring relationships may vary depending on the youth’s developmental context, prior attachment experiences, and readiness to trust adults after maltreatment. Youth who have experienced abuse often develop adaptive wariness toward adults, which may limit their openness or emotional investment in forming natural mentoring relationships (Neil et al., 2022). In such cases, natural mentors may offer companionship or limited support without fully addressing underlying attachment disruptions or providing the targeted coping and skill-building that buffer against depression. Prior research similarly suggests that while natural mentoring relationships can have broad and positive effects on youth development, they are rarely sufficient on their own to meet the complex needs of youth facing significant adversity (DuBois and Silverthorn 2005a). DuBois and Silverthorn (2005a) emphasize that mentoring should be viewed as one component within a more comprehensive system of supports, integrated alongside evidence-based practices that address multifaceted challenges.

Additionally, the unstructured and self-selected nature of natural mentoring may mean that these relationships differ widely in quality and intensity. Some may offer consistent, emotionally attuned support, while others may remain superficial or inconsistent, conditions that might fail to provide the level of corrective experience needed to offset early adversity. Qualitative studies using interviews or focus groups could offer crucial insight into these nuances by capturing how youth interpret and experience these relationships, the kinds of support mentors actually provide, and under what conditions mentoring feels protective versus insufficient.

Taken together, these patterns suggest that structure and intentionality may be key elements in determining when and how mentoring relationships function as protective. While natural mentors often provide emotional support (e.g., Hurd & Zimmerman, 2010; Raposa & Hurd, 2021), they may lack the scaffolding and goal-directed components that enhance skill development and promote resilience. Perhaps, the instrumental activities (e.g., goal directed activity, structured skill building) that formal mentors and mentees engage in play a significant role in how protective the relationship is for youth. In fact, this was found to be true for school-based mentoring programs. School-based mentors who had a close relationship with their mentee and engaged in instrumental activities had the largest positive impact on youth academic and behavior outcomes, compared to a close relationship alone (Lyons et al., 2019). Natural mentoring relationships differ significantly from formal mentoring relationships, in that they are flexible, unstructured, and usually unmonitored. Natural mentors may not engage in instrumental activities, which may in turn be impacting their salience as a protective factor for youth. In this case, researchers and policymakers may want to address child maltreatment through population-based approaches in addition to individual prevention efforts, such as mentoring. For example, researchers found that delivering evidence-based parenting interventions (i.e., Triple P, Positive Parenting Program) at the population-level resulted in decreased child maltreatment, with large effect sizes (*d* = 1.09 to 1.22), when compared to the control (Prinz et al., 2009).

Nevertheless, natural mentoring relationships are associated with positive youth outcomes, such as social-emotional development and academic and vocational functioning (Van Dam et al., 2018). Youth who experience childhood abuse can still receive some of these benefits from mentoring relationships, even if their mentors do not completely mitigate the depressive symptoms associated with childhood abuse. Future research should focus on deciphering the benefits of natural mentoring relationships for those who experience childhood abuse and the nuances that lie within those benefits (e.g., when natural mentoring is and is not a protective factor, impact of contextual factors). More research is needed to understand the interaction between supportive relationships and childhood abuse, and how to best support youth who experience childhood abuse.

### Limitations

Measurement limitations exist due to secondary data analysis. First, our understanding of childhood abuse was limited by the fact that emotional abuse was not included in Add Health. Emotional abuse is a common type of childhood abuse, along with physical abuse, sexual abuse, and neglect (Child Welfare Information Gateway, 2022). However, researchers and society at large have historically neglected to include emotional abuse in the conversation (Kumari, 2020). This may be partially explained by its lack of visibility (i.e., bodily injury) and the cultural differences (e.g., what constitutes emotional abuse) associated with it (Kumari, 2020). Despite challenges in identifying and measuring childhood emotional abuse, global estimates indicate that it is the most prevalent form of childhood maltreatment, affecting approximately 36% of individuals compared to lower rates for other abuse types (8–18%; Kumari, 2020). Moreover, emotional abuse frequently co-occurs with other forms of maltreatment, with overlap rates ranging from 62 to 95% (Shin et al., 2015). The lack of information on childhood emotional abuse in our current sample created a narrow understanding of childhood abuse, which may have impacted our results. Future research should replicate the current study using childhood abuse data that includes emotional abuse and determine whether there are differences in this relationship for emotional abuse compared to other forms of childhood abuse.

Second, some of the study variables of interest (i.e., presence of a natural mentor, life satisfaction) were measured using single-item questions, limiting the extent to which we could comprehensively understand these variables. Participants indicated whether they did or did not have a natural mentor (i.e., yes or no). Our analysis was limited to this question due to our focus on the presence or absence of a natural mentor. However, other items in Add Health ask about mentees’ perceptions of the mentoring relationship (e.g., importance, closeness) and the nature of the relationship (e.g., how the relationship was formed, frequency of contact, age of meeting). Many of these factors (e.g., quality, duration), beyond the mere presence of a natural mentor, have been found as important indicators for positive youth outcomes (DuBois and Silverthorn 2005b; Rhodes et al. 2006; Van Dam et al. 2018). Likewise, these mentoring relationship factors may influence the prominence of mentors as a protective factor for youth. Additionally, the use of a single-item measure to assess life satisfaction may not fully capture the multidimensional nature of this construct. Single-item measures can oversimplify complex psychological states and may not provide a comprehensive understanding of participants’ overall life satisfaction. While brevity can be important in survey design, future research should use well-validated measures when examining the role of natural mentors in the relationship between childhood abuse, life satisfaction, and depression to enhance reliability and validity of study findings.

Lastly, we were unable to establish temporal precedence due to the waves at which our variables of interest were collected in Add Health. As stated previously, childhood abuse, life satisfaction, and presence of a natural mentor were all collected at Wave III, and depression was collected at Wave IV. Given that the presence of a natural mentor was collected in the same wave as childhood abuse, there is no temporal precedence between childhood abuse and natural mentor. In other words, it remains unclear whether an individual had a natural mentor before or after experiencing childhood abuse. Future research should consider the timing and duration of the natural mentoring relationship to gain a more nuanced understanding of its potential as a protective factor for youth.

## Conclusion

Childhood abuse exerts profound effects on individuals’ mental health and well-being, manifesting lower life satisfaction and increased depressive symptoms in adulthood (Tanaka et al., 2015; Lindert et al., 2014). This study examined the influence of various forms of childhood abuse on these outcomes using a large, nationally representative dataset from Add Health, providing valuable insights into the broader population in the United States. Contrary to our hypothesis, the presence of a natural mentor did not mitigate the negative impact of childhood abuse on life satisfaction or depression. This finding challenges the notion that naturally occurring mentoring relationships buffer against adverse effects of trauma (Greeson, 2013; Southwick et al., 2007). Despite this, our findings underscore the critical need for further research into how different types of childhood abuse interact with natural mentoring relationships. Such insights are crucial for developing targeted interventions aimed at enhancing the resilience and well-being of those who have experienced childhood abuse. Additionally, understanding how different types of childhood abuse interact with various forms of social support, including natural mentoring, can inform effective prevention, early intervention, and treatment strategies. By addressing these gaps in knowledge, practitioners and policymakers can better support vulnerable populations to promote resilience in the face of childhood adversity.

## Data Availability

The current study used Add Health’s public-use data sets. Information on how to obtain these data sets is available on the Add Health website (https://addhealth.cpc.unc.edu/*).*
