# Correction to: Examining the Role of Natural Mentors in the Relationship Between Childhood Abuse, Life Satisfaction, and Depression: Findings From Add Health

**DOI:** 10.1007/s40653-026-00899-y

**Published:** 2026-04-30

**Authors:** Savannah B. Simpson, Bailey R. Dow, Samuel D. McQuillin

**Affiliations:** Department of Psychology, University of South Carolina, 1512 Pendleton Street, Barnwell College, Columbia, SC 29208 USA

**Correction to: Journal of Child & Adolescent Trauma (2026)**

10.1007/s40653-026-00873-8

The original online version of this article was revised. The author name, ‘Savannah Simpson,’ is duplicative. The correct author order should be: Savannah B. Simpson, Bailey R. Dow, & Samuel D. McQuillin. The last author, ‘Savannah Simpson,’ should be removed from the article.

The online version of the original article can be found at 10.1007/s40653-026-00873-8.

